# The HPV Serology Laboratory leads an initiative to standardize and harmonize human papillomavirus serology assays

**DOI:** 10.1371/journal.ppat.1011403

**Published:** 2023-06-29

**Authors:** Isabel Park, Troy J. Kemp, Ligia A. Pinto

**Affiliations:** HPV Serology Laboratory, Frederick National Laboratory for Cancer Research, Frederick, Maryland, United States of America; University of Arizona, UNITED STATES

## Abstract

The HPV Serology Laboratory is leading a global partnership initiative aiming for standardization and harmonization of current serology assay platforms being used to assess immune responses to HPV vaccines. Serology standardization is particularly important given the increasing number of immunobridging trials relying on serology data for approval of new vaccine dosing schedules or vaccine formulations. The initiative was established in 2017 to enable comparisons of data between different vaccines and relevant studies as well as expedite the implementation of new vaccines and vaccine indications. The HPV Serology Laboratory has held or attended several meetings with partnering laboratories, including international meetings in 2017, 2018, and 2021.

## Manuscript

To date, the licensed human papillomavirus (HPV) prophylactic vaccines have demonstrated robust immunogenicity and efficacy [1–4]. Although immune correlates of protection against HPV infection are unclear due to the exceptional efficacy of the vaccines, a growing body of preclinical and clinical data suggest HPV-specific neutralizing antibodies as the primary mechanism of protection [5–8]. Thus, HPV serology measurements are being proposed as endpoints in clinical trials evaluating immunogenicity and efficacy of modified regimens of the existing vaccines and follow-on products. Unfortunately, until recently there was a lack of standardization of assays, protocols, and reagents widely available and accessible to the scientific community for the assessment of immune responses to the vaccines.

Building on serology assay standardization and harmonization efforts initiated by the World Health Organization (WHO), HPV LabNet in 2006, a joint meeting with several institutions, organized by Ligia Pinto from Frederick National Laboratory for Cancer Research (FNLCR) and Elizabeth Unger from Centers for Disease Control and Prevention (CDC), was held in March 2016 to collectively recognize the growing need for standardization and harmonization. The participants of the meeting included the Bill & Melinda Gates Foundation, NCI, CDC, United States Food and Drug Administration (FDA), WHO, GlaxoSmithKline (GSK), Merck, Innovax, National Institute for Biological Standards and Control (NIBSC), Public Health England, and several US and international universities and research institutes.

Briefly, in response to the initial convention, the HPV Serology Laboratory (HPVSL) at FNLCR was established in January 2017 to address this challenge, in partnership with the National Cancer Institute and the Bill & Melinda Gates Foundation. A follow up meeting at the 32nd International Papillomavirus Conference in Sydney, Australia from October 2 to 6, 2018, was convened to discuss the progress of main aims, challenges, and some of the future needs. The outcomes of this meeting included definition of the primary mission of the initiative, which was to enable comparisons of data between different vaccines and different relevant studies, as well as expedite the implementation of new vaccines and vaccine indications. The specific aims to drive these goals include:

1. The development of a qualified secondary assay standard and a bank of serum specimens to use as assay proficiency panels for 9 HPV types (HPV-6/11/16/18/31/33/45/52/58) included in the licensed HPV vaccines.
2. The production of qualified reference reagents (HPV Virus-Like Particles) by establishing a set of criteria or guidelines for qualification.
3. The development of a validated multiplex serology assay to enable immunogenicity monitoring in HPV vaccine trials.
4. The promotion of the use of standards through platforms such as meetings, publications, data, and protocol sharing.

We herein briefly describe the progress to each of the 4 objectives as well as provide the links to all currently developed standard operating procedures (SOPs).

First, HPVSL was able to generate qualified secondary standards calibrated against HPV-16 and HPV-18 WHO International Standards (IS). For this effort, 144 human serum samples were obtained from various sample providers, and each sample was screened and categorized depending on the specific antibody response for each HPV type. Moreover, an HPV assay proficiency panel of 80 samples covering a wide range of HPV antibody responses was established. Currently, the proficiency panel has been distributed and tested by 10 laboratories with their respective antibody binding or pseudovirion-based neutralization assays.

Second, the production of the first lot of qualified VLPs for multiple HPV types (HPV-6/11/16/18/31/33/45/52/58) was completed at the time of the follow up meeting in 2018. These reagents will be available for reference use in assays in respective laboratories.

Third, a high-throughput Luminex bead-based 9-plex immunoassay was developed and a validation plan for the HPV-16 and HPV-18 ELISAs and multiplex assay was drafted. To date, the 9-plex assay validation has been completed and guidance documents for data processing and analyses are under development.

Lastly, efforts have been made to promote the development and use of standards, SOPs, and data (Table 1). Up to 8 material transfer agreements had been established at the time of the follow up meeting in 2018. Protocol and data sharing with the greater HPV serology community are now enabled by a dedicated FNL HPV Serology Laboratory website, where investigators and researchers can access serology laboratory SOPs, guidance documents for VLP production and qualification, and multiplex assay validation protocol.

**Table 1:** List of HPV Serology Laboratory standard operating procedures documents and corresponding forms.

	Title	Version
10023	Good Documentation Practices	1.0
15006	Reagent Preparation	4.0
* Forms*:	* *15006–01_Reagent Preparation* *15006–02_HPV Plate Coating Form*	4.0
15010	Equipment Qualification and Calibration	2.0
* Forms*:	*15010–01*: *Equipment Calibration Documentation Form*	
15013	Rounding and Significant Figures	1.0
20000	Bacterial Culture Maintenance	3.0
* Forms*:	*20000–01_Bacterial Plasmid Culture Form*	
20001	HEK293TT Cell Culturing and Maintenance	7.0
* Forms*:	*20001–02_293TT Cell Thaw Form* *20001–03_Cell Culture Maintenance Form* *20001–04_293TT Cell Culture Freezing Form*	
20002	Preparation of Bacterial Glycerol Stock for Plasmid Purification	1.0
* Forms*:	*20002–01_Preparation of Bacterial Glycerol Stock for Plasmid Purification Form*	
20003	Procedure for Separating Serum and Plasma from Whole Blood	1.0
* Forms*:	*20003–01_Serum and Plasma Separation Procedure Form*	
20005	Plasmid DNA Transfection in HEK293TT for VLP Production and Purification	5.0
* Forms*:	20005–01: HEK293TT Transfection Form, Day 1–4 20005–02: HEK293 Transfection Form, Day 20005–03: Fraction Pool Form	
20006	Ripcord pDNA Transfection in HEK293TT for Pseudoviruses Production and Purification	1.0
* Forms*:	*20006–01_HEK293TT Transfection Form*, *Day 1–4* *20006–02_HEK293TT Transfection Form*, *Day 5* *20006–03_Fraction Pool Form*	
20007	Bacterial Transformation	1.0
20010	Plasmid Purification Using a Zymo Research Kit	1.0
* Forms*:	*20010–01_ZymoPure II Maxiprep Kit Plasmid Purification Form*	
20011	HPV VLP Conjugation to Luminex-Based Microspheres	1.0
* Forms*:	*HPV VLP Coupling to Luminex Magnetic Beads Data Capture Form*	
26000	Biosafety Cabinet (BSC) Use and Maintenance	2.0
* Forms*:	*26000–01_BSC Daily Use Form* *26000–02_BSC Maintenance Form*	
26001	Operation, Use and Maintenance of CO2 Incubators	3.0
* Forms*:	*26001–01_CO2 Incubator Weekly Maintenance Form* *26001–02_CO2 Incubator Maintenance form*	
26002	Use and Maintenance of a BioTek Plate Washer	4.0
* Forms*:	*26002–01_Microplate Washer Carryover Check Form* *26002–02_Plate Washer Maintenance Form* *26002–03*: *Plate Washer Daily Use Maintenance Form*	
26003	Use and Maintenance of a Molecular Devices M5 Plate Reader	3.0
* Forms*:	*26003–01_Molecular Devices Plate Reader Monthly Maintenance Form* *26003–02_Molecular Devices Plate Reader Plate Calibration Form*	
26004	Use and Maintenance of the Cellometer Cell Counter	4.0
* Forms*:	*26004–01_Cellometer Monthly Maintenance Form* *26004–02_Cellometer Monthly Quality Check Form*	
26005	Use and Maintenance of a 2-8C Refrigerator	4.0
* Forms*:	*26005–01_Rrefrigerator Maintenance Form* *26005–02_Rrefrigerator Temperature Monitor Log Form*	
26006	Use and Maintenance of the Liquid Nitrogen Freezer	3.0
* Forms*:	*26006–01_Liquid Nitrogen Freezer Maintenance Form*	
26007	Use and Maintenance of the Water Bath	2.0
* Forms*:	*26007–01_Waterbath Monthly Maintenance Form*	
26008	Use and Maintenance of Incubator Shakers	2.0
* Forms*:	*26008–01_Maintenance of Incubator Shaker Form*	
26009	Use and Maintenance of Pipettes	1.0
* Forms*:	*26009–01_Pipette Accuracy Confirmation Form*	
26010	Use and Maintenance of a Thermomixer	4.0
* Forms*:	*26010–01_Thermomixer Maintenance Form*	
26011	Use and Maintenance of a pH Meter	3.0
* Forms*:	*26011–01_pH Daily Use Calibration Form* *26011–02_pH Maintenance Form*	
26012	Use and Maintenance of an Analytical & Precision Balance	3.0
* Forms*:	*26012–01_Analytical Balance Maintenance Form* *26012–02_Precision Balance Maintenance Form*	
26013	Use and Maintenance of -20C Freezers	3.0
* Forms*:	*26013–01_-20°C Freezer Maintenance Form* *26013–02_-20°C Freezer Temperature Monitor Log Form*	
26014	Use and Maintenance of a Laboratory Convection Incubators	3.0
* Forms*:	*26014–01_Convection Incubator Maintenance Form* *26014–02_Convection Incubator Weekly Maintenance Form*	
26015	Use and Maintenance of an Inverted Microscope	3.0
* Forms*:	*26015–01_Inverted Microscope Use and Maintenance Form*	
26016	Operation, Use and Maintenance of the Water Purification Systems	1.0
* Forms*:	*26016–01_Maintenance of the Water Purification Systems Form*	
26017	Use and Maintenance of the Eppendorf microcentrifuge	3.0
* Forms*:	*26017–01_Eppendorf Microcentrifuge Maintenance Form*	
26018	Use and Maintenance of NanoDrop Spectrophotometers	3.0
* Forms*:	*26018–01_ NanoDrop 1000 Spectrophotometer Maintenance* *26018–02_ NanoDrop One c Spectrophotometer Maintenance Form* *26018–03_NanoDrop Spectrophotometer Calibration Check Form*	
26019	Controlled-Rate Cell Freezing Using a CoolCell Device	2.0
* Forms*:	*26019–01_CoolCell Maintenance Form*	
26020	Use and Maintenance of a Microplate Shaker	3.0
* Forms*:	*26020–01_Microplate Shaker Maintenance Form*	
26021	Use and Maintenance of the Optima XPN Ultracentrifuge System	2.0
* Forms*:	*26021–01_Optima XPN Ultracentrifuge Use Form* *26021–02_Optima XPN Ultracentrifuge Maintenance Form*	
26022	Use and Maintenance of the Combination Refrigerator and Freezer	2.0
* Forms*:	*26022–01_Combination Refrigerator and Freezer Use and Maintenance Form*	
26023	Use and Maintenance of the Agilent Bioanalyzer	2.0
* Forms*:	*26023–01_Bioanalyzer Maintenance Form*	
26024	FlexMap Use and Maintenance_v. 1.0	1.0
* Forms*:	*26024–01_Use and Weekly Maintenance of the FLEXMAP 3D* *26024–02_FLEXMAP 3D Performance Maintenance Form*	
26025	Use and Maintenance of LI-COR Imager	1.0
* Forms*:	*26025–01_LI-COR Maintenance Form*	
26027	Use and Maintenance of the FYRITE Gas Analyzer	1.0
* Forms*:	*26027–01_Maintenance of the FYRITE Gas Analyzer Form*	
26028	General Use and Maintenance of the MESO QuickPlex SQ 120 Instrument	1.0
* Forms*:	*26028–01_MESO QuickPlex SQ 120 Instrument Monthly Maintenance Form*	
26029	Verification and Use of Electronic Thermometer	1.0
* Forms*:	*26029–01_Thermometer Maintenance Form*	
26030	Use and Maintenance of -80C Freezers	3.0
* Forms*:	*26030–01_-80C Freezer Maintenance Form*	
26031	epMotion 96 Use and Maintenance	1.0
* Forms*:	*26031–01_epMotion 96 Maintenance Form*	
26033	Use and Maintenance of the Thermo Fisher Sorvall XTR Centrifuge	4.0
* Forms*:	*26033–01_Sorvall XTR Centrifuge Maintenance Form*	
26034	General Use and Maintenance of a Fume Hood	1.0
* Forms*:	*26034–01_Fume Hood Maintenance Form*	
26035	Verification and Use of Laboratory Timers	1.0
* Forms*:	*26035–01_Timer Calibration Verification Form*	2.0
26037	Use and Maintenance of the Gel Tank and Power Supply_v.1.0_Employee	1.0
* Forms*:	*Gel Tank and Power Supply Maintenance Form*	
30000	HPV Neutralization Assay for Titer Determination	5.0
* Forms*:	*30000–01_HPV Neutralization Assay*, *Sample Preparation Form* *30000–02_HPV Neutralization Assay*, *Substrate Development Form* *30000–03_HPV Neutralization Assay*, *Titer Substrate Development Form*	
30001	HPV Antibody ELISA	4.0
* Forms*:	*30001–01_HPV_Antibody_ELISA_Data_Capture_Form* *30001–02_HPV_Standard_Preparation_Form*	
30002	HPV-16 and HPV-18 Avidity ELISA	1.0
	HPV Avidity ELISA Form	1.0
30009	BCA Protein Assay	4.0
* Forms*:	*30009–01_BCA Data Capture Form*	
30010	Acrylamide Protein Gel Analysis of HPV Virus-Like Particles (VLPs)	6.0
* Forms*:	*30010–01*: *Acrylamide Protein Gel Data Capture Form*	
30011	Protein Analysis of Virus-Like Particles (VLPs) Using the Agilent 2100 Bioanalyzer	3.0
* Forms*:	*30011–01_Bioanalyzer Data Capture Form*	
30012	ELISA to Determine VLP Specificity	3.1
* Forms*:	*30012–01_ELISA to Determine VLP Specificity Data Capture Form*	
30013	HPV Multiplex Assay_Version 1.0	1.0
* Forms*:	*30013–01_HPV Multiplex Assay Data Capture Form*	
40000	Identity and Access Management for LabVantage(IAM)	3.0
* Forms*:	*40000–01_ LIMS_Request_for_System_Access_Form*	
40001	Introduction to LabVantage	1.0
40002	Performing HPV ELISA Testing Using LES Worksheet	1.0
40005	Prepare Mixed Consumables Using LES Worksheet	2.0
40006	Receiving and Managing Consumables	1.0
40007	Receiving and Managing Samples in LIMS	2.0
* Forms*:	*40007–01_Sample Receipt Form*	
40008	Performing Plasmid DNA Production using LES Worksheet	1.0
40009	Performing Cell Maintenance using LES Worksheet	1.0
40010	Performing Plasmid DNA Transfection and VLP Purification using LES Worksheet	1.0
40011	Performing VLP Fraction analysis using LES Worksheet	1.0
40012	Performing VLP Fraction Pooling and final VLP lot analysis using LES Worksheet	1.0

*All forms are included in the above SOP and are provided for standardized documentation of experimental conditions and results.

All documents are available upon request and all hyperlinked/underlined SOPs are available at our website (https://frederick.cancer.gov/research/vaccine-immunity-and-cancer-directorate/hpv-serology-laboratory).

As part of this initiative, the HPVSL has been collaborating with NIBSC and WHO to develop International Standards for the remaining HPV types in the nonavalent vaccine, including HPV high-risk types (HPV-31, HPV-33, HPV-45, HPV-52, and HPV-58) and low-risk types (HPV-6 and HPV-11). These standards were recently approved by WHO and are now available for request through NIBSC’s website (https://www.nibsc.org/products/brm_product_catalogue/sub_category_listing.aspx?category=Vaccines&subcategory=Human%20Papillomavirus).

Future directions discussed by the initiative consisted of (1) implementation of high-throughput HPV antibody testing using Good Clinical Laboratory Practices (GCLP) to support ongoing and future vaccine immunogenicity trials; and (2) development of an HPV Vaccine Trial Network of laboratories involved in the testing of HPV vaccines to expand training capability as well as establish an Assay Proficiency Panel Program.

In June 2021, a virtual meeting was held on June 29 to 30, 2021, to review progress of the standardization initiative thus far and to bridge scientific gaps and outstanding questions. The main aims and outcomes of the meeting were to discuss: (1) standardization of assays and reagents; (2) International Standard Calibration procedures; (3) assay cut-off values; (4) current immunobridging clinical trials; (5) gaps and challenges in standardization of HPV serology. A total of 119 scientists and clinicians from 13 countries, representing 36 institutions and industries, participated in the meeting to update the community on the progress on the objectives of the initiative. Participants also reported on advancements made by the HPV serology community in exploring and advancing with new vaccine dosing schedules or vaccine formulations. A report summarizing the meeting has been recently published [9].
